# *MedScrubCrew*: A Medical Multi-Agent Framework for Automating Appointment Scheduling Based on Patient-Provider Profile Resource Matching

**DOI:** 10.3390/healthcare13141649

**Published:** 2025-07-08

**Authors:** Jose M. Ruiz Mejia, Danda B. Rawat

**Affiliations:** Department of Electrical Engineering and Computer Science, Howard University, Washington, DC 20059, USA

**Keywords:** clinical decision support system, large language model, multi-agent system, agentic workflows, Gale-Shapley Matching, patient-provider profiles

## Abstract

**Background:** With advancements in Generative Artificial Intelligence, various industries have made substantial efforts to integrate this technology to enhance the efficiency and effectiveness of existing processes or identify potential weaknesses. Context, however, remains a crucial factor in leveraging intelligence, especially in high-stakes sectors such as healthcare, where contextual understanding can lead to life-changing outcomes. **Objective:** This research aims to develop a practical medical multi-agent system framework capable of automating appointment scheduling and triage classification, thus improving operational efficiency in healthcare settings. **Methods:** We present *MedScrubCrew*, a multi-agent framework integrating established technologies: Gale-Shapley stable matching algorithm for optimal patient-provider allocation, knowledge graphs for semantic compatibility profiling, and specialized large language model-based agents. The framework is designed to emulate the collaborative decision making processes typical of medical teams. **Results:** Our evaluation demonstrates that combining these components within a cohesive multi-agent architecture substantially enhances operational efficiency, task completeness, and contextual relevance in healthcare scheduling workflows. **Conclusions:** *MedScrubCrew* provides a practical, implementable blueprint for healthcare automation, addressing significant inefficiencies in real-world appointment scheduling and patient triage scenarios.

## 1. Introduction

The ever-increasing complexity of healthcare presents a critical need for effective automation solutions to alleviate some of the profession’s most time-consuming tasks, such as triaging in emergency room or family practice. Although considered a rudimentary task, triaging remains a persistent challenge across medical facilities in various domains. This challenge became particularly evident during the COVID-19 crisis, when medical resources, such as equipment, personnel, and time, were pushed to their limits. During this period, healthcare providers were often required to make rapid, high-stakes decisions to save lives and deliver optimal medical care to those in need.

Medical professionals must always make swift and informed decisions about their patients’ health. In certain cases, these decisions may have only a minor impact on the overall health of the patient. However, in other situations, they can significantly influence quality of care and, consequently, alter the expected outcomes of the patient. These decisions often depend on how medicine is practiced, which can vary significantly due to environmental factors and subjective interpretations. Among the many processes involved in patient care, triaging stands out as one of the most common yet contentious aspects.

In the medical context, triaging refers to the classification of patients based on the urgency of their needs, allowing the efficient allocation of time, personnel, and resources [1,2]. Although this may seem straightforward, it requires input from multiple areas and roles within medical crews to provide a truly relevant context for decision-making. In order to stay ahead of possible resource allocation, medical crews work together to forecast the prioritization of resources for a patient typically a day before by reviewing their schedule and discussing potential needs. This process is colloquially known as “scrubbing” the patient’s schedule [3]. This process becomes increasingly challenging when resources are scarce and time is limited. Implementing a multi-agent system with specialized knowledge in these respective areas involved in patient treatment improves visibility, enabling the better identification of potential treatment routes and more effective resource allocation [4].

This research makes the following system-level contributions:

- We develop *MedScrubCrew*, a multi-agent system framework operationalizing the collaborative roles of a medical crew through specialized LLM-based agents.
- We integrate knowledge graphs to model and retrieve semantically compatible patient-provider profiles.
- We implement Gale-Shapley stable matching within this framework to allocate patients to providers based on resource availability and compatibility.
- We conduct simulation-based evaluations on task completion, tool correctness, and contextual relevancy, demonstrating the feasibility of orchestrating such frameworks in real-world healthcare settings.

Together, these contributions address critical resource-dependent inefficiencies in healthcare systems, offering strong potential for real-world implementation and future innovation.

The structure of this paper is as follows: Section 1 provides an introduction to our research. Section 2 elaborates on the gaps in recent research on medical multi-agent systems. Section 3 presents background information on state-of-the-art components in multi-agent systems. Section 4 describes the dataset and methodologies used in this framework. Section 5 elaborates on the theoretical and experimental multi-agent design. Section 6 discusses the evaluation metrics. Section 7 summarizes the results from the experiment. Section 8 provides a discussion and limitations. Section 9 provides our conclusion. Appendix A offers the conclusion.

## 2. Related Works

Recent advancements in LLM-based frameworks and multi-agent systems have aimed to enhance healthcare reasoning, triage decision-making, and diagnostic accuracy. Tang et al. [5] proposed MEDAGENTS, a multi-agent collaborative reasoning framework utilizing role-playing LLM agents to improve the medical question-answering performance in zero-shot settings. While demonstrating improved QA accuracy compared to chain-of-thought prompting, MEDAGENTS is limited to single QA tasks without addressing operational challenges such as patient-provider scheduling, triage workflows, or resource allocation. Additionally, it lacks traditional scheduling or triage systems, does not integrate knowledge graphs for semantic compatibility profiling, and omits complexity analysis for deployment feasibility.

Schmidgall et al. [6] introduced AgentClinic, a multimodal agent benchmark simulating clinical environments to evaluate LLM diagnostic reasoning under interactive settings, biases, and multilingual constraints. However, AgentClinic is purely a benchmarking platform focused on evaluating diagnostic decision-making accuracy. It does not implement scheduling, triage matching, or resource optimization frameworks, and it does not integrate knowledge graphs or matching algorithms for operational systems. Its emphasis remains on agent evaluation rather than system implementation for healthcare workflows.

Han and Choi [7] developed a multi-agent CDSS integrating LLM agents for KTAS-based triage classification and treatment planning in emergency departments. Their system demonstrated improved triage classification accuracy by emulating ED staff roles, such as triage nurse, physicians, and coordinators. Nevertheless, it is confined to triage-level classification within KTAS protocols [8] and does not address appointment scheduling or patient-provider matching. Moreover, their framework does not employ stable matching algorithms for optimal resource allocation and lacks integration with knowledge graphs for semantic retrieval, limiting explainability and operational optimization. It also does not consider dynamic rescheduling or real-time resource optimization.

Other researchers have integrated the Korean Triage and Acuity Scale (KTAS) [8] for the assessment of triage and the RxNorm API in medication management. This research highlights the potential of LLM-driven multi-agent CDSS in improving emergency care management, reducing ED overcrowding, and improving patient outcomes.

Overall, while prior works focus on diagnostic reasoning [5] and benchmarking agentic performance [6] or triage classification [7], none integrate agentic LLM reasoning, knowledge graphs for semantic compatibility profiling, and stable matching algorithms into a single framework for operational appointment scheduling and triage workflows. Additionally, existing works lack traditional scheduling or triage heuristics, complexity analysis for scalability, and system-level evaluation.

In this work, we present *MedScrubCrew*, a practical multi-agent medical scheduling framework that integrates stable matching, knowledge graphs, and role-based LLM agents to automate appointment scheduling and triage classification. Our system addresses these gaps by demonstrating an orchestrated solution for real-world healthcare operations.

## 3. Brief Overview of Multi-Agent System for Healthcare

### 3.1. Agentic Artificial Intelligence (Agentic AI)

Agentic AI refers to AI systems designed to act autonomously by independently analyzing information, making informed decisions, and performing tasks in complex environments, thus enhancing operational efficiency in various domains [9]. In recent years, the pursuit of Artificial General Intelligence (AGI) has sparked discussions about leveraging specialized agents in specific fields to improve performance and outcomes.

As noted in [10], their study emphasizes collaboration with subject matter experts, identifying real-time scenarios, and acquiring relevant knowledge to optimize agent functionality. In the healthcare domain, agents can assume roles in specialized medical areas, potentially reducing context learning errors that might arise with general-purpose LLMs [4,10]. This specialization enables agents to focus on domain-specific tasks, improving accuracy and reliability in critical decision-making processes.

### 3.2. Agentic Prompt Engineering

Prompt engineering involves carefully designing input prompts to guide AI models in generating desired outputs, thus improving the precision and relevance of their responses [11]. This approach is similarly applied to agents that are instances of the same or different LLMs. However, agents’ prompts are tailored differently because they include additional information or context that helps agents understand their purpose and develop a chain of thought to determine the best way to achieve their goals. These tailored prompts typically consist of a role, goal, and background to facilitate an agent’s in-context learning.

### 3.3. In-Context Learning

In-context learning allows AI models to adapt to new tasks by utilizing information provided within the input context, enabling flexible and dynamic responses without the need for extensive retraining. According to recent studies [12,13], in-context learning does not require weight updates, training pipelines, and, in some cases, prompting in a few shots. It simply involves gathering inference from previous inputs and the current input context.

In [14], the authors claimed that in-context learning is prompt-dependent. As long as the prompt is aligned with the data it is trying to learn, the model can establish contextual patterns. In [13], the authors present how in-context learning can allow scene understanding, which is something LLM tends to struggle with due to trained knowledge. However, even with a large context window, some of the context may become skewed or lost during the generation process. Due to this sensitivity to the potential loss of context during learning or generation, agentic systems have gained prominence for their ability to preprocess data and retrieve relevant high-quality inputs for in-context learning.

## 4. Dataset and Proposed Method

In this section, we focus on identifying a high-quality dataset and developing a framework that simulates an electronic health record (EHR) system that is suitable for modeling semantic relationships.

In this section, we describe the system implementation of *MedScrubCrew*, detailing how we integrate existing components such as knowledge graphs, LLM-based specialized agents, and Gale-Shapley stable matching into a unified framework to automate medical appointment scheduling and triage classification. This approach aims to closely replicate the processes of a medical crew. In a recent study [15], the authors created a graphical clinical database that simulates an EHR together with metadata consisting of a holistic profile of the patient, the provider, and the medical facility.

### 4.1. Medical Information Mart for Intensive Care IV (MIMIC-IV)

MIMIC-IV database [16] is widely utilized and publicly accessible database that offers comprehensive clinical data from intensive care units, supporting a range of medical research and machine learning projects.

#### 4.1.1. Patient Dataset

The patient dataset contains detailed demographic and clinical information, facilitating the comprehensive analyses of patient characteristics and health outcomes across different populations [17].

#### 4.1.2. Triage Dataset

The triage dataset contains data related to the initial assessment and prioritization of patients, which are essential for evaluating and enhancing triage procedures in healthcare settings [16].

#### 4.1.3. Admission Dataset

The admission dataset records information about patient admissions, including admission times, reasons for hospitalization, and associated medical interventions, aiding studies on hospital utilization and patient flow [17].

#### 4.1.4. Notes Dataset

The notes dataset comprises clinical documentation and physician notes, providing qualitative insights into patient care, treatment plans, and medical observations [18].

#### 4.1.5. Vital Signs Dataset

The vital signs dataset includes the time-series measurements of patients’ vital statistics, such as heart rate, blood pressure, and temperature, crucial for monitoring patient health and predicting clinical outcomes [16].

### 4.2. Multi-Agent Components

The multi-agent components serve as the foundation for agents to collaborate and delegate tasks effectively. The system integrates several specialized AI agents, each assigned distinct roles to collaboratively manage various aspects of the healthcare workflow, mimicking the functionality of a medical crew [19]. This is achieved by providing specific and actionable tasks utilizing advance tools.

#### 4.2.1. Tasks

The tasks managed by the multi-agent system are designed to replicate real-world medical processes, including scheduling appointments, classifying triage levels, and managing patient data effectively. Table 1 presents the details of the tasks and how they are structured for utilization in agentic systems.

#### 4.2.2. Tools

Tools are specialized functions that allow an agent to complete certain tasks, such as searching the web, generating an image, or interacting with certain files. Table 2 describes the arsenal of tools available for an agentic system. The benefit of using tools is that they are attached to agents but are not necessarily used if tasks can be completed and the goal can be achieved without them.

#### 4.2.3. Hierarchical Process

The hierarchical process outlines the structured workflow of the multi-agent system, detailing how tasks are prioritized, delegated, and coordinated among different agent levels to ensure efficient operation [20]. This process allows for an agent to assume the role of management, providing delegation and collaboration between other agents. It is useful for review and revision-dependent tasks.

### 4.3. Knowledge Graph

The knowledge graph integrates various sources of patient data, establishing semantic relationships between entities to support informed decision-making and effective patient-provider matching [21]. Semantics enable the system to interpret and use medical data accurately, while entities such as patients, healthcare providers, and medical conditions form the core of the graph structure. Relationships illustrate connections between entities, such as patient-provider links or treatment associations, creating a cohesive healthcare ecosystem [22]. The system retrieves and manipulates data efficiently, enabling complex queries and comprehensive analysis.

In [15], the authors employed a hybrid natural language processing approach using a fine-tuned DeBERTa model (decoding-enhanced BERT with disentangled attention) trained to perform named entity recognition tasks. Subsequently, they utilized a generative LLM to establish relationships between the identified entities. This approach was shown to be effective in facilitating research on the creation of patient-provider profiles.

The database agent uses a pre-populate knowledge graph containing 999 nodes and 2387 relationships from a 100-patient sample. It contains nodes ranging from demographics, medical facilities, biomarkers, pharmaceutical, and past medical history, among others [15].

### 4.4. Gale-Shapley Stable Matching

In this research, we applied stable matching to optimize patient-provider profile matching based on available resources and the services of both sides. We decided to select the Gale-Shapley algorithm because it allows for stable matching, meaning that matching is carried out not according to best preferences but according to the optimal choice [23]. In a scenario where a patient does not have all their preferences matched, they can still have the optimal choice. As shown in Algorithm 1, this allows priority to be assigned to the provider’s schedule if the patient meets the criteria set by the provider.
**Algorithm 1** Gale-Shapley Matching Algorithm  1:INPUT: *U*: set of unmatched patients; *P*: set of patients; *R*: set of providers; S(p,r): compatibility score function  2:OUTPUT: *M*, a matching of patients to providers  3:Initialize U←P  4:Initialize M(p)←⌀,∀p∈P  5:Initialize Π(p)←⌀,∀p∈P  6:**while **U≠⌀** do**  7:      **for** p∈U  **do**  8:       $r\leftarrow \mathrm{highest}-\mathrm{ranked}\;r^{\prime }\in R\setminus \Pi \left(p\right)$  9:      Π(p)←Π(p)∪{r}10:      **if** $M^{-1}\left(r\right)=⌀$ **then**11:        M(p)←r12:        U←U∖{p}13:      **else**14:        Let $\tilde{p}$ be the current match of *r*15:        Compute rank(p,r) and $\mathrm{rank}(\tilde{p},r)$ in *r*’s preference list16:        Compute scores $S_{p}=S\left(p,r\right)$ and $S_{\tilde{p}}=S\left(\tilde{p},r\right)$17:         **if** $\mathrm{rank}\left(p,r\right)<\mathrm{rank}\left(\tilde{p},r\right)\;\vee \;\left(\mathrm{rank}\left(p,r\right)=\mathrm{rank}\left(\tilde{p},r\right)\wedge S_{p}>S_{q}\right)$ **then**18:          M(q)←⌀19:          $U\leftarrow U\cup \{\tilde{p}\}$20:          M(p)←r21:          U←U∖{p}22:         **end if**23:      **end if**24:    **end for**25:**end while**26:**return** *M*

In [24], the authors addressed the problem of patient-trial matching using patient records and clinical trial eligibility criteria through multiclass classification. Similarly, this research leverages a large language model (LLM) to process and integrate the outputs of various agents, using semantic similarity to calculate a compatibility score for each criterion.

Other studies [25,26] focused on the scenario of patients providing proposals to providers or medical facilities; however, in emergent situations, medical crews have the need to allocate appropriate resources depending on multiple factors that have little relevance to the health of patients but rather the ability of the medical crew to perform.

The Gale-Shapley algorithm facilitates the optimal matching of nodes in a bipartite graph [27,28]. However, because provider and patient characteristics can change due to new patients, updated patient criteria, or hourly adjustments in provider resources, it is essential that the nodes are able to dynamically adapt to these changes. This ensures that the matching process continues to achieve optimal results based on the updated preferences. We provide an example output in Appendix A, Listing A1.

### 4.5. Automated Machine Learning

Automated machine learning (AutoML) techniques are applied to streamline the selection and optimization of models, improving the predictive capabilities of a system with minimal manual intervention. In this study, we selected regression models to determine the triage level for vital signs and measured these models using the mean absolute error, mean squared error, and the coefficient of determination, and we performed cross-validation with a fold of 5 to correctly predict the level of acuity, as seen in Table 3. In Table 4, we provide details of the hyperparameter used for the top three contending models. We utilized a list of regression models, such as Linear Regression, Ridge Regression, Lasso Regression, Random Forest Regressor, Gradient Boosting Regressor, AdaBoost Regressor, Bagging Regressor, Extra Trees Regressor, and HistGradientBoosting Regressor. See Appendix A, Table A1, for additional model metrics.

#### 4.5.1. HistGradientBoosting Regressor

After evaluating the scoring of the models, we observed that the HistGradientBoosting Regressor model achieved satisfactory scores for the assignment, as seen in Table 3. The model can be mathematically represented as follows:

(1) $${\hat{y}}_{i}=\sum_{m=1}^{M}\eta \cdot h_{m}\left(x_{i}\right)$$

where

- ${\hat{y}}_{i}$ is the predicted value for the *i*-th observation;
- *M* is the total number of iterations (or weak learners);
- η is the learning rate, controlling the contribution of each weak learner;
- $h_{m}\left(x_{i}\right)$ is the prediction of the *m*-th weak learner for input $x_{i}$.

The HistGradientBoosting Regressor builds an ensemble of weak learners, typically decision trees, with each learner trained to minimize the residual errors of the preceding ensemble [29]. Its histogram-based optimization efficiently handles large datasets by discretizing continuous features into bins, enabling faster computation and reduced memory usage.

#### 4.5.2. Mean Absolute Error

In the context of the regression model, the mean absolute error (MAE) quantifies the average magnitude of errors between the predicted acuity levels and the actual acuity levels without considering their direction. It is calculated as follows:

(2) $$\mathrm{MAE}=\frac{1}{n}\sum_{i=1}^{n}\left|y_{i}-{\hat{y}}_{i}\right|$$

where

- *n* is the total number of observations;
- $y_{i}$ is the actual acuity level for the *i*-th observation;
- ${\hat{y}}_{i}$ is the predicted acuity level for the *i*-th observation.

A lower MAE indicates that the regression model’s predictions are closer to the actual acuity levels on average, making it particularly useful for understanding the overall accuracy of the model in clinical settings [30].

#### 4.5.3. Mean Squared Error

The mean squared error (MSE) evaluates the average squared difference between the predicted acuity levels and the actual acuity levels. It penalizes larger errors more than smaller ones, making it more sensitive to outliers. The formula for MSE is as follows:

(3) $$\mathrm{MSE}=\frac{1}{n}\sum_{i=1}^{n}{\left(y_{i}-{\hat{y}}_{i}\right)}^{2}$$

where

- *n* is the total number of observations;
- $y_{i}$ is the actual acuity level for the *i*-th observation;
- ${\hat{y}}_{i}$ is the predicted acuity level for the *i*-th observation.

In the AutoML Regression Model, a lower MSE reflects smaller average errors in predicting acuity levels, but the metric’s sensitivity to outliers should be considered when interpreting the results [31].

#### 4.5.4. Coefficient of Determination ($R^{2}$)

The coefficient of determination ($R^{2}$) measures how well the predicted acuity levels explain the variance in the actual acuity levels. It is calculated as follows:

(4) $$R^{2}=1-\frac{\sum_{i=1}^{n}{\left(y_{i}-{\hat{y}}_{i}\right)}^{2}}{\sum_{i=1}^{n}{\left(y_{i}-\bar{y}\right)}^{2}}$$

where

- $y_{i}$ is the actual acuity level for the *i*-th observation;
- ${\hat{y}}_{i}$ is the predicted acuity level for the *i*-th observation;
- $\bar{y}$ is the mean acuity level across all observations;
- $\sum_{i=1}^{n}{\left(y_{i}-{\hat{y}}_{i}\right)}^{2}$ is the residual sum of squares (RSS);
- $\sum_{i=1}^{n}{\left(y_{i}-\bar{y}\right)}^{2}$ is the total sum of squares (TSS).

For the AutoML Regression Model, an $R^{2}$ value close to 1 indicates that the tool’s predictions explain most of the variance in acuity levels, while a value close to 0 suggests limited predictive power [32,33]. This metric is particularly valuable for evaluating the overall effectiveness of the model in clinical decision-making.

**Figure 1:**
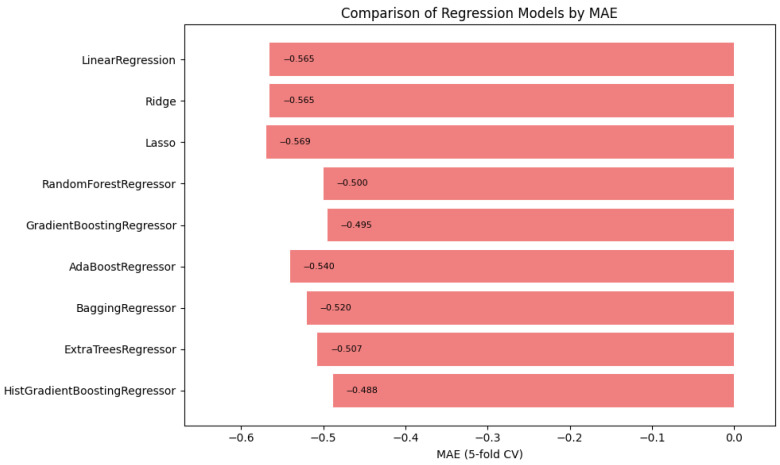
Automated regression MAE score: a bar plot showing the mean absolute error (MAE) scores across different regression models.

**Figure 2:**
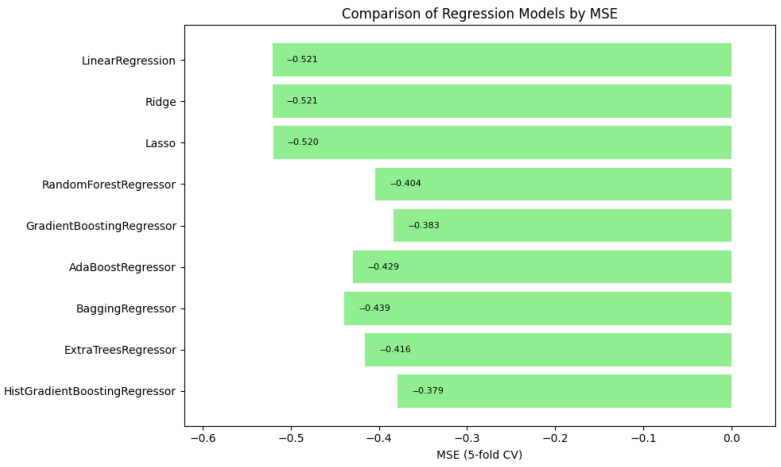
Automated regression MSE score: a bar plot showing the mean squared error (MSE) scores across different regression models.

**Figure 3:**
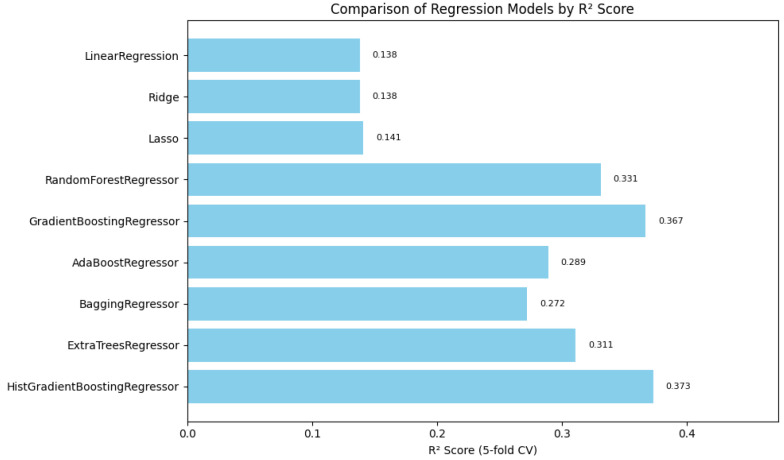
Automated regression $R^{2}$ score: a bar plot showing the $R^{2}$ scores across different regression models, representing the proportion of variance explained by each model.

In summary, Figure 1, Figure 2 and Figure 3 illustrate the comparative performance of various regression models evaluated in predicting the patient acuity levels. Figure 1 presents the HistGradientBoosting Regressor, which achieved the lowest MAE of approximately 0.488, suggesting it generates the most precise predictions compared to other evaluated models. Figure 2 shows again the HistGradientBoosting Regressor delivered the best performance with an MSE of around 0.379, demonstrating robustness in minimizing substantial prediction errors. It is important to note that while these metrics were recorded as negative values due to scikit-learn scoring convention, they are reported here in their absolute form. Lastly, Figure 3 illustrates $R^{2}$ in which the model attained the highest score of approximately 0.0373, indicating its superior ability to capture the underlying patterns within the data. Collectively, these metrics underscore the HistGradientBoosting Regressor as the appropriate model, providing robust and accurate predictions for patient triage acuity within the presented automated machine learning framework.

## 5. Medical Multi-Agent Experimental System Design

**Figure 4:**
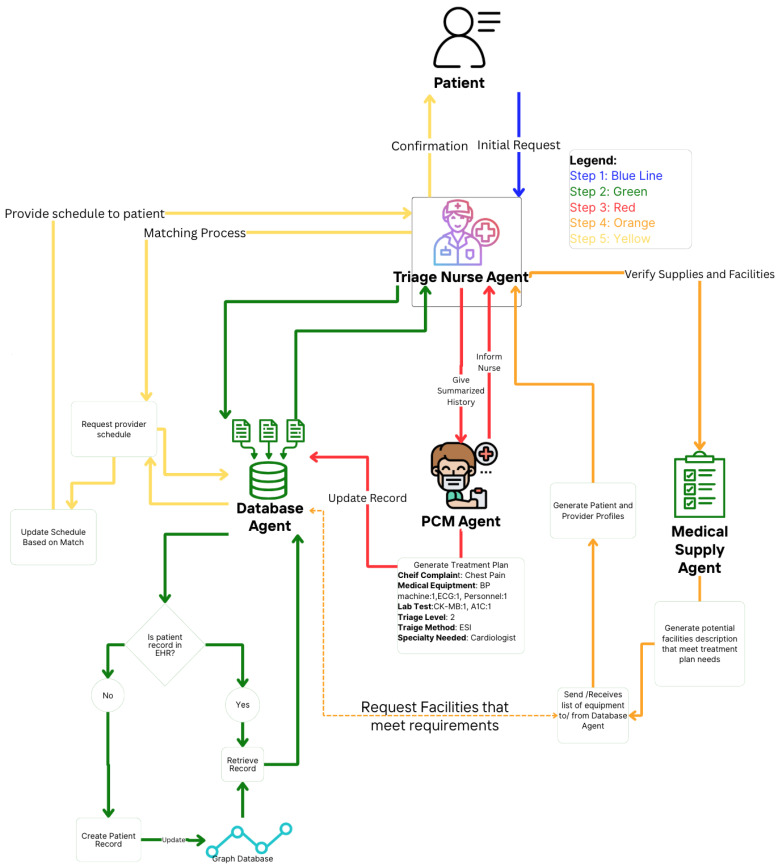
Medical multi-agent design: A schematic representation of the multi-agent system used for medical processes. Patient information is hypothetical.

This section proposes a theoretical and experimental design in a multi-agent medical system. In Figure 4, an automated approach is shown to gather relevant patient data based on prognoses conditioned from the initial complaints. The agents collaborate in order to create the patient-provider profile utilizing LLM to interpret in-context learning from each agent output [10]. Table 1 provides descriptions of the tasks for the specialized agents.

### 5.1. Patient Scheduling and Triage Process

We modeled the patient scheduling and triage process as a discrete-time-state machine that follows a set of protocols typically used to re-assess and increase visibility on a patient’s status [34]. According to [35], to automate an EHR, it must follow a set of processes that resembles the manual process a medical professional undergoes in order to achieve a proper schedule and triage status. The state machine governing this process explicitly accounts for new patient requests and involves a sequence of agent-performed tasks. Let S(t) denote the system’s state at the time *t*, which captures the current patient schedule. Let R(t) be a new patient request arriving at time *t*, or R(t)=NoReq if no request arrives. By convention, each subroutine ${F\;}_{*}\left(S,\mathrm{NoReq}\right)$ simply propagates *S* unchanged. Thus, we define the following:

(5) $$S\left(t+1\right)\;=\;\left\{\begin{array}{cc} F\left(S(t),\;R(t)\right),\; & \mathrm{if}\;R(t)\neq \mathrm{NoReq},\\ F\left(S(t),\;\mathrm{NoReq}\right),\; & \mathrm{otherwise} \end{array}\right.$$

(6) $$F\left(S,R\right)\;=\;\left(F_{\mathrm{appointment}}\;\circ \;F_{\mathrm{logistic}}\;\circ \;F_{\mathrm{prognosis}}\;\circ \;F_{\mathrm{profile}}\;\circ \;F_{\mathrm{vital}}\;\circ \;F_{\mathrm{history}}\right)\left(S,R\right).$$

In Equation (6), this composition operates from the right to the left, beginning with the evaluation of the patient’s history and culminating in appointment scheduling.

**The following are defined:**

S(t):       Current state of the system at time *t*.
S(t+1):   Next state of the system at time t+1.
R(t):       New patient request submitted at time *t* (if any).
F(S,R):    Composite function representing the sequence of agent tasks:
  - $F_{\mathrm{history}}\left(S,R\right)$: Triage nurse agent assesses patient history based on *S* and *R*.
  - $F_{\mathrm{vital}}\left(\cdot \right)$: Triage nurse agent evaluates new vital signs.
  - $F_{\mathrm{profile}}\left(\cdot \right)$: Primary care provider agent creates the patient’s illness profile.
  - $F_{\mathrm{prognosis}}\left(\cdot \right)$: Primary care provider agent develops the prognosis plan with required resources.
  - $F_{\mathrm{logistic}}\left(\cdot \right)$: Medical logistic agent reviews the resource plan and identifies the top three providers with available resources.
  - $F_{\mathrm{appointment}}\left(\cdot \right)$: Appointment scheduling agent matches patient-provider profiles and schedules the appointment.
  ⌀ represents the absence of a new patient request.

### 5.2. Database Agent

A database agent specializes in retrieving and updating the graphical knowledge EHR database. This type of agent functions as a retrieval-augmented generation component, providing context for the framework and supporting the goals of other agents [36,37]. It leverages an instance of a LLM to generate valuable patient insights and enhance search queries by translating natural language into database-specific languages, such as Cypher [38,39].

### 5.3. Primary Care Provider Agent

The primary care provider (PCP) agent assumes the role of a PCP, which can be a physician or nurse practitioner with general medical knowledge in clinical medicine. PCPs are responsible for maintaining a patient’s overall health [40,41]. The goal of the PCP agent is to accurately assign a prognosis and propose a potential treatment plan, which can then be used to create the patient profile.

### 5.4. Medical Logistic Agent

The goal of the medical logistic agent is to perform semantic comparisons between the preferences and characteristics of the patient profile and the provider profile. It collaborates with the database agent to query updated medical logistics, including resources, time, personnel, and provider services [42,43,44]. We acknowledge that semantic differences may arise based on facility-specific terminology and resource naming conventions. For instance, cardiologists may use different terms for equipment or services that are functionally similar.

To address this, the medical logistic agent is specialized in medical logistics, medical services, and equipment terminology, enabling it to accurately compare terms semantically. Additionally, it structures the patient-provider profile as the expected output, ensuring compatibility and alignment between patient needs and provider capabilities.

### 5.5. Triage Nurse

The triage nurse acts as the manager agent in this hierarchical framework. A triage nurse can be described as the nurse in charge of the patient process. In contrast to the PCP agent, which is responsible for the prognosis of illness and the services needed, the triage nurse is responsible for the overall process, which involves patient EHR review, resource allocation, and appointment scheduling [45]. This is portrayed in Figure 4, where the patient-provider profiles is given to triage nurse agent to assign a scheduling slot. In addition to reviewing, it delegates tasks such as retrieving historical information or creating a new patient inquiry.

### 5.6. Profile-Based Compatibility

To support patient-provider matching, we define a numerical compatibility score that quantifies how well a patient’s characteristics align with those of a provider. Let $\pi_{p}$ and $\pi_{r}$ denote the structural profiles of the patient *p* and provider, respectively. These profiles may include attributes such as the medical condition, urgency, medical equipment needed, or specialty. The resulting score reflects how well a provider matches a patient’s needs.

We define the compatibility score using an abstract function *f*:

(7) $$S\left(p,r\right)=f\left(\pi_{p},\;\pi_{r}\right),$$

where *f* is a domain-specific function that returns a real-valued compatibility score based on the alignment between retrieved patient and provider profiles. While the implementation details of *f* are abstracted in this work, it may represent a learned scoring model or a rule-based similarity metric tailored to the medical triage and scheduling context. The profiles may be composed of structured features:

$$\pi_{p}=\left({\mathrm{condition}}_{p},\;{\mathrm{urgency}}_{p},\;{\mathrm{location}}_{p},\ldots \right),\;\pi_{r}=\left({\mathrm{specialty}}_{r},\;{\mathrm{capacity}}_{r},\;{\mathrm{location}}_{r},\ldots \right).$$

Higher values of S(p,r) represent better compatibility between the two. The similarity function *f* may be implemented using cosine similarity, a dot product, or a trained neural metric.

To convert these scores into ordinal preferences, we define patient and provider rankings based on sorted compatibility scores. Specifically, for each patient *p*, the rank of provider *r* is defined as follows:

(8) $${\mathrm{rank}}_{p}\left(r\right)=1+\left|\{\;r^{\prime }\in R:S\left(p,r^{\prime }\right)>S\left(p,r\right)\}\right|,$$

Similarly, for each provider *r*, the rank of patient *p* is as follows:  

(9) $${\mathrm{rank}}_{r}\left(p\right)=1+\left|\{\;p^{\prime }\in P:S\left(p^{\prime },r\right)>S\left(p,r\right)\}\right|.$$

In this formulation, a rank of 1 indicates the most preferred match, and ties are implicitly handled via strict inequalities in the set definitions.

### 5.7. Patient-Provider Profile Matching Process

The patient-provider matching process is executed using the Gale-Shapley matching algorithm. We first match each patient in *P* to a provider in *R* based on mutually ranked preferences. Before the algorithms begin, every patient is unmatched, and no proposals are made. For each p∈P, we set the match and proposal sets as follows:

(10) M(p)=⌀,Π(p)=⌀,∀p∈P

At each iteration, an unmatched patient makes proposals to its most preferred provider, which has not yet been approached. We let ${\mathrm{rank}}_{p}\left(r^{\prime }\right)$ denote the position of provider $r^{\prime }$ in patient *p*’s preferences list, with 1 indicating the highest preferred choice. The next provider to receive a proposal is given by   

(11) $$r=\arg\min_{r^{\prime }\in R\setminus \Pi \left(p\right)}\left({\mathrm{rank}}_{p}\left(r^{\prime }\right)\right)$$

which ensures that *p* always makes proposals to the highest ranked available provider.

When a patient *p* proposes to provider *r*, *r* compares *p* against its current match $\tilde{p}$. If *r* prefers *p* over $\tilde{p}$, then *p* becomes matched to $\tilde{p}$; otherwise, *p* remains unmatched. This update rule is expressed as

(12) $$M\left(p\right)=\left\{\begin{array}{cc} r, & \mathrm{if}\;\mathrm{provider}\;r\;\mathrm{prefers}\;\mathrm{patient}\;p\;\mathrm{over}\;\mathrm{its}\;\mathrm{current}\;\mathrm{match}\;\tilde{p}\\ ⌀, & \mathrm{otherwise}. \end{array}\right.$$

To ensure stable matching so that no patient-provider pair will prefer each other over their assigned matches, we enforce the following acceptance and rejection criteria. A provider *r* only accepts a proposal from *p* if *r* prefers *p* to any alternative $r^{\prime }$ that *p* might have preferred, and *r* rejects an existing match $\tilde{p}$ in favor of *p* only if $p{≻}_{r}\tilde{p}$:

(13) $$\begin{array}{cc} r{≻}_{p}r^{\prime } & \Rightarrow M(p)=r\;\mathrm{only}\;\mathrm{if}\;\mathrm{provider}\;r\;\mathrm{accepts}\;\mathrm{patient}\;p \end{array}$$

(14) $$\begin{array}{cc} p{≻}_{r}\tilde{p} & \Rightarrow \mathrm{provider}\;r\;\mathrm{rejects}\;\mathrm{patient}\;\tilde{p}\;\mathrm{in}\;\mathrm{favor}\;\mathrm{of}\;\mathrm{patient}\;p \end{array}$$

Finally, the matching algorithm terminates after at most n·m proposals because each of the n=|P| patients can make proposals to at most m=|R| providers.

The computation complexity of the Gale-Shapley matching algorithm utilized in *MedScrubCrew* is O(nm), where *n* is the number of patients and *m* is the number of providers. This is because, in the worst-case scenario, each patient may make proposals to every provider before finding a stable match. For typical scheduling scenarios involving hundreds of patients and providers, the algorithm remains computationally feasible, with runtime increasing linearly with the number of proposals. Additionally, since *MedScrubCrew* integrates dynamic patient and provider updates, periodic recomputation may be required. However, the deterministic nature of the algorithms and the complexity of the polynomial time ensure that scheduling can be executed efficiently within the practical operational time frame for daily appointment allocation tasks in clinical settings.

(15) TotalProposals≤n·m.

Table 5 provides a description of the notation used in this matching process.

## 6. Evaluation and Metrics

During this research, we employed an LLM for the Judge method. In essence, the LLM-as-a-Judge framework utilizes an LLM instance to evaluate responses based on predefined metrics [46]. We evaluated the agents’ responses based on the following criteria: the context provided (if any), the correct selection of tools, and task completeness.

### 6.1. Contextual Relevancy

Some tasks were provided with context in the form of previous agent responses. This allows the multi-agent system to maintain contextual awareness throughout the process. This metric calculates contextual relevancy by taking the number of relevant statements and dividing it by the total number of generated statements produced by the LLM. The resulting score ranges from 0, meaning no relevant context, to 1, meaning the response fully meets the relevant contextual criteria:

(16) $$CR=\frac{N_{\mathrm{relevant}}}{N_{\mathrm{total}}},$$

where CR represents the contextual relevancy score, $N_{\mathrm{relevant}}$ is the number of relevant statements, and $N_{\mathrm{total}}$ is the total number of statements generated, as seen in Appendix A, Listing A2.

The contextual relevance evaluation is defined as follows:

(17) $$M\left(C\right)=\left\{\begin{array}{cc} 1 & \mathrm{if}\;CR\geq \tau ,\\ 0 & \mathrm{otherwise}, \end{array}\right.$$

where M(C) represents whether the response maintains contextual relevancy, and τ is the predefined threshold.

The preference condition for maintaining context is given by the following:

(18) $$C_{\mathrm{relevant}}≻C_{\mathrm{irrelevant}}\Rightarrow M\left(C\right)=1\;\mathrm{only}\;\mathrm{if}\;\mathrm{context}\;\mathrm{is}\;\mathrm{maintained},$$

(19) $$C_{\mathrm{incorrect}}≺C_{\mathrm{correct}}\Rightarrow C_{\mathrm{incorrect}}\;\mathrm{is}\;\mathrm{refined}\;\mathrm{to}\;\mathrm{improve}\;\mathrm{relevancy},$$

where $C_{\mathrm{relevant}}≻C_{\mathrm{irrelevant}}$ means that the relevant context is preferred, and $C_{\mathrm{incorrect}}≺C_{\mathrm{correct}}$ means that the incorrect context is adjusted.

The termination condition for contextual evaluation is as follows:

(20) TotalContextEvaluations≤k·m,

where *k* is the number of statements per evaluation, and *m* is the number of iterations performed.

### 6.2. Task Completeness

In a multi-agent system, each agent is assigned a variety of tasks. In some cases, a task may not be fully completed based on the expected input and output. The system evaluates whether the task description aligns with the generated output. This metric is based on binary scoring and assesses how closely the generated output satisfies the given task requirements.

(21) TCS=AlignmentScore(T,O),

where TCS represents the task completion score, T is the assigned task, and O is the generated output.

The task completion evaluation is defined as follows:

(22) $$M\left(T\right)=\left\{\begin{array}{cc} 1, & \mathrm{if}\;\mathrm{AlignmentScore}(T,O)\geq \tau ,\\ 0, & \mathrm{otherwise}, \end{array}\right.$$

where M(T) represents whether a task T is successfully completed, and τ is the threshold for considering a task as complete.

The preference criterion for task alignment is given by the following:

(23) $$\begin{array}{cc} T_{\mathrm{completed}} & ≻T_{\mathrm{incomplete}}\Rightarrow M\left(T\right)=1\;\mathrm{only}\;\mathrm{if}\;O=T, \end{array}$$

(24) $$\begin{array}{cc} T_{\mathrm{incorrect}} & ≺T_{\mathrm{correct}}\Rightarrow T_{\mathrm{incorrect}}\;\mathrm{is}\;\mathrm{refined}\;\mathrm{until}\;\mathrm{completion}, \end{array}$$

where $T_{\mathrm{completed}}≻T_{\mathrm{incomplete}}$ denotes that a completed task is preferred over an incomplete one, and $T_{\mathrm{incorrect}}≺T_{\mathrm{correct}}$ signifies that an incorrect task undergoes refinement until completion.

The termination of the task execution process is guaranteed as follows:

(25) TotalTaskAttempts≤k·n,

where *k* is the number of tasks, and *n* represents the number of iterations required for task completion. We present an example prompt for how it calculates the scores in Appendix A, Listing A3.

### 6.3. Tool Correctness

Agents can be provided a multitude of tools in order to complete their tasks. These tools can range from a calculator to web searches. However, in some cases, the agent might have either similar tools that provide different responses or a wide variety of tools. In this metric, the tool selection will be evaluated to see if the selection is correct for the actual tool indicated:   

(26) $$TC=\frac{N_{\mathrm{correct}}}{N_{\mathrm{total}}},$$

where TC represents tool correctness, $N_{\mathrm{correct}}$ represents the number of correctly used tools (or correct input parameters/outputs), and $N_{\mathrm{total}}$ represents the total number of tools used.

The tool selection evaluation is defined as follows:

(27) $$M\left(T\right)=\left\{\begin{array}{cc} 1 & \mathrm{if}\;T\;\mathrm{is}\;\mathrm{used}\;\mathrm{correctly},\\ 0 & \mathrm{otherwise}, \end{array}\right.$$

where M(T) represents whether a tool *T* is used correctly.

The correctness criterion for tool usage is given by the following:

(28) $$T_{\mathrm{selected}}≻T_{\mathrm{alternative}}\Rightarrow M\left(T\right)=1\;\mathrm{only}\;\mathrm{if}\;T\;\mathrm{is}\;\mathrm{used}\;\mathrm{correctly},$$

(29) $$T_{\mathrm{incorrect}}≺T_{\mathrm{correct}}\Rightarrow T_{\mathrm{incorrect}}\;\mathrm{is}\;\mathrm{discarded}\;\mathrm{in}\;\mathrm{favor}\;\mathrm{of}\;T_{\mathrm{correct}},$$

where $T_{\mathrm{selected}}≻T_{\mathrm{alternative}}$ means that the selected tool is preferred over an alternative, and $T_{\mathrm{incorrect}}≺T_{\mathrm{correct}}$ means that an incorrect tool is replaced with the correct one.

The termination of the tool selection process is ensured as follows:

(30) TotalToolEvaluations≤k·m,

where *k* is the number of tasks, and *m* is the number of tools. The accuracy of the $T_{\mathrm{correct}}$ and $T_{\mathrm{incorrect}}$ values in the tool correctness metric is ensured through a deterministic and objective evaluation of expected tools using exact matching by the tool’s name and, optionally, by input parameters and $T_{\mathrm{correct}}$ if it fully aligns with these expectations; any tool that does not meet these criteria is counted as $T_{\mathrm{incorrect}}$. This rule-based approach leaves no room for ambiguity or the subjective reflection of the number of correct usages, ensuring that the TC metric is a precise and reliable measure of agent tool selection and performance.

## 7. Results

Multi-agent systems enable a wide range of applications. However, understanding their behaviors and decision-making processes is essential for improving explainability, particularly in justifying their generated responses. For the GPT-4o-mini [47] model, the evaluation was conducted using its own instance, while all other models were evaluated using GPT-4o-mini due to its lower latency and faster response times for smaller evaluation tasks.

**Figure 5:**
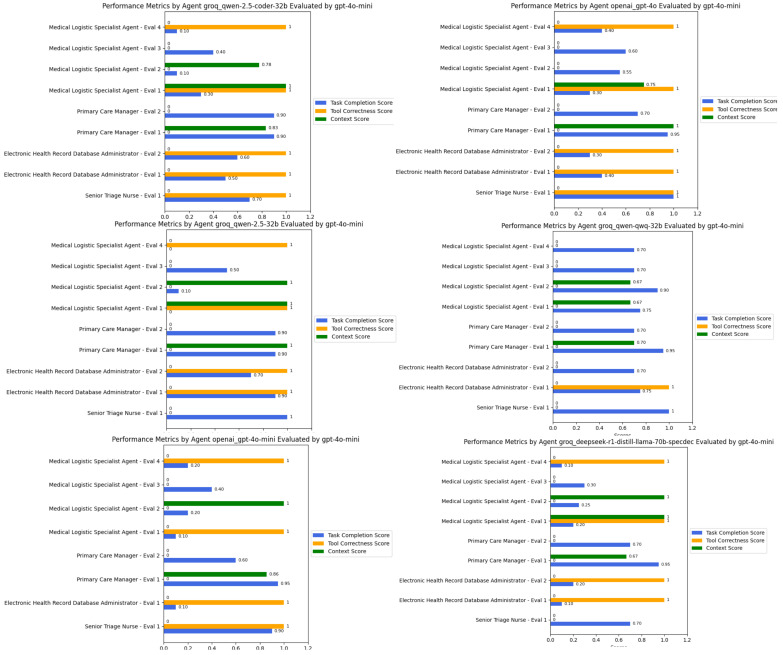
Performance comparison of six state-of-the-art large language models with function-calling capabilities across three evaluation metrics: contextual relevancy, tool usage accuracy, and task completion rate. The QWEN-2.5-coder-32b model demonstrates consistent suitable performance across all metrics with an overall average score of approximate 0.71.

We utilized six state-of-the-art models as the engines that power these agents. The results were evaluated based on contextual relevancy, tool usage, and task completion. As shown in Figure 5, our observations indicate that QWEN-2.5-coder-32b effectively selected and utilized the provided tools to generate appropriate responses while maintaining context. For instance, in the triage assessment task, the following was the case.

In contrast, the other Qwen models exhibited inconsistent performance. While Qwen-2.5-32B was able to retrieve relevant EHR notes, its task completion scores fluctuated between 0.7 and 0.9, reflecting occasional misinterpretations of task objectives despite retrieving medically relevant data. However, its logistical decision-making performance was lower, as it often failed to complete medical logistics profiling tasks, resulting in lower effectiveness when structuring ICU requirements or streamlining supply allocation. Similarly, the DeepSeek-R1-Distilled model struggled in retrieving EHR data, scoring 0.1 in some retrieval tasks, largely due to syntax errors in graph database queries.

Interestingly, in the medical logistical matching task, all models experienced errors during tool execution, often due to improper input formatting or missing expected attributes even with a structured Pydantic input/output model. This underscores a broader challenge in medical logistics optimization, where model precision in structured query generation directly impacts system effectiveness.

Overall, QWEN-2.5-coder-32b demonstrated superior adaptability, particularly in decision-making tasks where it had access to well-structured tools. However, areas requiring deeper contextual synthesis, such as long-form EHR retrieval, highlighted incomplete data extraction across all models, suggesting the need for enhanced retrieval-augmented generation (RAG) capabilities for improved medical decision support.

## 8. Discussion and Limitations

*MedScrubCrew* demonstrates the feasibility of implementing a practical multi-agent system combining coordination, compatibility-based stable matching, and semantic knowledge graphs. While it does not propose new algorithms, it provides a deployment-focused framework addressing operational inefficiencies in scheduling and triage workflows. In our experiments, Agent collaboration was occasionally disrupted by API rate-limit throttling and delayed tool calls, compromising responsiveness at scale. Lightweight reasoning models sometimes bypass specialized tool calls for trivial triaging tasks, still yielding correct outcomes via full-profile LLM processing but introducing potential bias by neglecting targeted, rule-based checks. Detailed agent collaboration also rapidly exhausts the LLM context window, increasing latency and risking the loss of earlier conversation states.

We also recognize the importance of prompt engineering in agentic workflows in providing clear and substantive role and task descriptions. To advance our research, we plan to implement an AI feedback loop to enhance the accuracy of generated outputs and review decisions made by the triage nurse during the scheduling of patient appointments.

Furthermore, we acknowledge several factors requiring further exploration: using other evaluation metrics such as Hallucination, Bias, and Prompt Alignment; the availability of medical testing results prior to scheduling; and the completion of referrals before appointment allocation. These features can significantly influence the position of a patient on the schedule and require additional considerations in the experimental design.

We also acknowledge the importance of security and privacy in a production environment. The data used is an open-source de-identified dataset that was used to create the needed information for the multi-agent crew to function. However, in a production environment, security and privacy would have to be taken into account, such as having self-hosted models, contractual agreements with LLM providers to self-host a specific model, or simply having guardrails in place for input verification.

While our evaluation tasks are structured and deterministic, future work will incorporate human clinician validation to complement LLM as a Judge evaluation and mitigate potential scoring biases.

Beyond these technical challenges, our framework omits several real-world considerations. We have not yet incorporated human-in-the-loop oversight. Clinicians cannot intervene during matching, which is critical for safety and trust. Additionally, dynamic resource constraints (e.g., fluctuating staff availability), telehealth scenarios, and interactions with IoT-enabled diagnostic devices remain unexplored.

## 9. Conclusions

In this research, we presented *MedScrubCrew*, an applied multi-agent system framework integrating specialized LLM agents, knowledge graphs, and stable matching to automate appointment scheduling and triage classification. While building on existing algorithms and AI tools, our work demonstrates practical system integration for healthcare automation. Future work will focus on clinician-in-the-loop evaluations and real-world pilot studies to assess clinical utility.

## Figures and Tables

**Table 1:** Agentic tasks.

Task Name	Prompt Description	Expected Output	Agent
triage_assessment_task	Determine triage method: use ESI (Levels 1–5) for large hospitals; START (Green, Yellow, Red, and Black) for smaller clinics.	Selected triage level.	Triage Nurse Agent
pcm_prognosis_task	Given triage level and chief complaint, produce a prognosis with outcomes and treatment recommendations.	Prognosis plan (explanation, treatment options, care recommendations).	Primary Care Manager
pcm_resource_task	List required medical resources (labs, exams, equipment) for patient care.	Detailed resource list.	Primary Care Manager
ehr_data_retrieval_task	Build a Neo4j Cypher query to fetch a patient’s EHR data (visit frequency, history, interventions) without extraneous nodes.	Summary of EHR data.	EHR Specialist
ehr_data_similarity_task	From retrieved EHR notes, find the one most similar to the patient’s chief complaint.	Comparative summary of best-matching note vs. complaint.	EHR Specialist
logistical_query_task	Generate a Cypher query to pull facility and provider info for a given healthcare setting.	Facility/provider details.	Medical Logistic Specialist
patient_profiling_task	Create a patient profile (needs, preferences, resources with quantities).	Structured patient-profile dictionary.	Medical Logistic Specialist
provider_profiling_task	Build a provider profile (specialties, availability, resources).	Structured provider-profile dictionary.	Medical Logistic Specialist
matching_task	Retrieve schedules from PostgreSQL and run Gale-Shapley to match patients and providers.	JSON of optimal patient-provider matching.	Medical Logistic Specialist

**Table 2:** Agentic medical process tools.

Tool Name	Description	Input Details
Neo4j Query Tool	Connects to a Neo4j database and executes one or multiple Cypher queries, enabling efficient extraction of medical information.	Query: A Cypher query string or list of strings;
		URI: Connection URI (defaults to NEO4J-URI);
		Username: Username (defaults to NEO4J-USERNAME);
		Password: Password (defaults to NEO4J-PASSWORD).
Auto Regression Tool	Performs regression using scikit-learn or XGBoost, either predicting from feature values or training on a dataset of medical records.	sbp: optional systolic blood pressure; pain: optional pain level;
		dbp: optional diastolic blood pressure; heart rate: optional;
		O_2_sat: optional oxygen saturation; resp. rate: optional;
		Temperature: optional body temperature; acuity: optional acuity level;
		Data: optional CSV path or DataFrame for training.
Gale–Shapley Matching Tool	Matches patients to providers via an applied Gale–Shapley algorithm, optimizing based on mutual preferences and compatibility scores.	Patients: {ID → (preferences list; characteristics dict)};
		Providers: {ID → (preferences list; characteristics dict)}.
Appointment Schedule Tool	Retrieves a provider’s current schedule, along with associated patient profiles.	Provider: Identifier for the provider; Date: Schedule date.

**Table 3:** Top three models by performance metrics.

Model	R^2^ (Mean)	MSE (Mean ± SE)	MAE (Mean ± SE)
HistGradientBoosting Regressor	0.373	−0.379±0.001	−0.488±0.001
GradientBoosting Regressor	0.367	−0.383±0.001	−0.495±0.000
RandomForest Regressor	0.331	−0.405±0.001	−0.500±0.001

**Table 4:** Hyperparameter settings for selected models.

Model	Hyperparameters
RandomForest Regressor	$n_{\mathrm{estimators}}=100,\;\mathrm{random}\_\mathrm{state}=42$
GradientBoosting Regressor	$n_{\mathrm{estimators}}=100,\;\mathrm{learning}\_\mathrm{state}=0.1$
HistGradientBoosting Regressor	max_iter=100

**Table 5:** Notations used in the Gale-Shapley matching algorithm.

Notation	Description
*P*	Set of patients with preferences and characteristics
*R*	Set of providers with preferences and characteristics
S(p,r)	Compatibility score function for patient *p* and provider *r*
*M*	Matching of patients to providers
*U*	Set of unmatched patients
M(p)	Current match for patient *p* (initially empty)
Π(p)	Set of providers patient *p* has proposed to
*r*	Provider under consideration during a patient’s proposal
*q*	Current patient matched to a provider
rank(p,r)	Position of patient *p* in provider *r*’s preferences
${\mathrm{rank}}_{p}\left(r\right)$	Position of provider *r* in patient *p*’s preference list
$S_{p}$	Compatibility score of patient *p* with provider *r*
$S_{\tilde{p}}$	Compatibility score of patient $\tilde{p}$ with provider *r*

## Data Availability

Data are contained within the article.

## Appendix A

**Listing A1.** Gale–Shapley output showing patient-provider matches with compatibility scores.

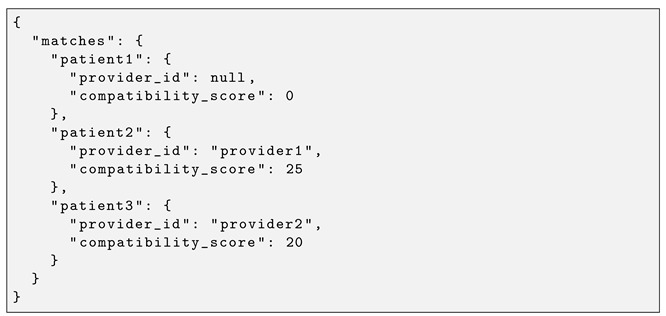

**Listing A2.** Prompt for context relevance evaluation.

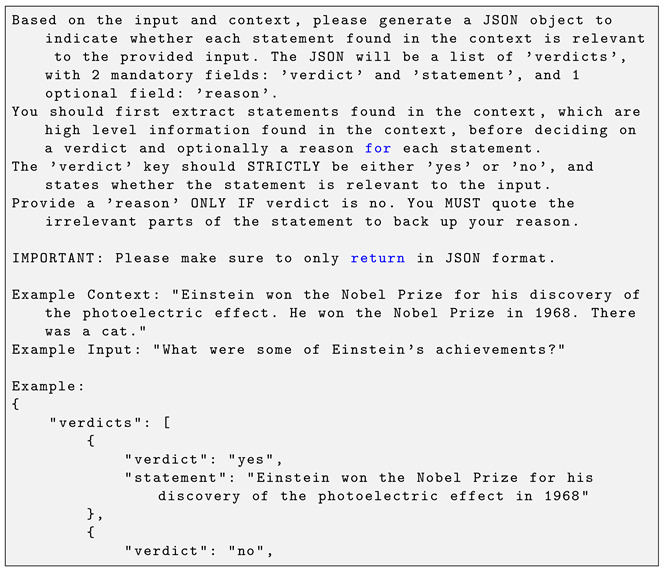
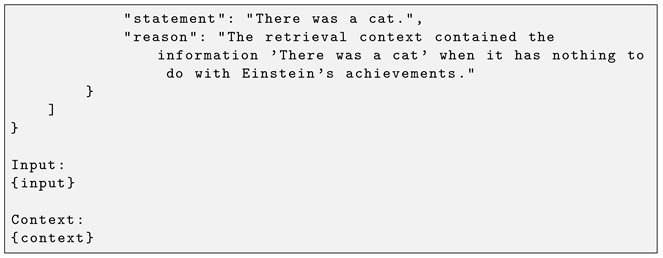

**Listing A3.** Prompt for task completeness evaluation.

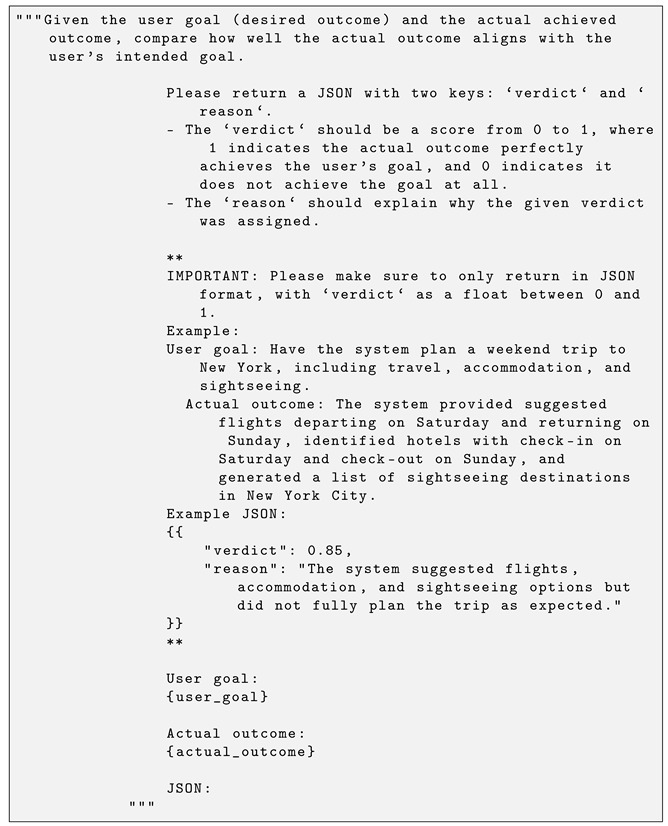

**Table A1:** Model evaluation metrics (mean and standard error) for all regressors.

Model	$R^{2}$	MSE		MAE	
	Mean	Mean	SE	Mean	SE
Linear Regression	0.138	−0.521	0.038	−0.565	0.001
Ridge	0.138	−0.521	0.038	−0.565	0.001
Lasso	0.141	−0.520	0.036	−0.569	0.001
RandomForest Regressor	0.331	−0.404	0.001	−0.500	0.000
GradientBoosting Regressor	0.367	−0.383	0.001	−0.495	0.000
AdaBoost Regressor	0.289	−0.429	0.002	−0.540	0.001
Bagging Regressor	0.272	−0.439	0.001	−0.520	0.000
ExtraTrees Regressor	0.311	−0.416	0.001	−0.507	0.001
